# Food and nutrient intakes and compliance with recommendations in school-aged children in Ireland: findings from the National Children’s Food Survey II (2017–2018) and changes since 2003–2004.

**DOI:** 10.1017/S0007114522002781

**Published:** 2023-06-14

**Authors:** Laura Kehoe, Maria Buffini, Breige A. McNulty, John M. Kearney, Albert Flynn, Janette Walton

**Affiliations:** 1 School of Food & Nutritional Sciences, University College Cork, Cork, Ireland; 2 UCD Institute of Food & Health, University College Dublin, Dublin, Ireland; 3 School of Biological & Health Sciences, Technological University Dublin, Dublin, Ireland; 4 Department of Biological Sciences, Munster Technological University, Cork, Ireland

**Keywords:** Food intake, Nutrient adequacy, School-aged children, Food-Based Dietary Guidelines

## Abstract

The childhood years represent a period of increased nutrient requirements during which a balanced diet is important to ensure optimal growth and development. The aim of this study was to examine food and nutrient intakes and compliance with recommendations in school-aged children in Ireland and to examine changes over time. Analyses were based on two National Children’s Food Surveys; NCFS (2003–2004) (*n* 594) and NCFS II (2017–2018) (*n* 600) which estimated food and nutrient intakes in nationally representative samples of children (5–12 years) using weighed food records (NCFS: 7-d; NCFS II: 4-d). This study found that nutrient intakes among school-aged children in Ireland are generally in compliance with recommendations; however, this population group have higher intakes of saturated fat, free sugars and salt, and lower intakes of dietary fibre than recommended. Furthermore, significant proportions have inadequate intakes of vitamin D, Ca, Fe and folate. Some of the key dietary changes that have occurred since the NCFS (2003–2004) include decreased intakes of sugar-sweetened beverages, fruit juice, milk and potatoes, and increased intakes of wholemeal/brown bread, high-fibre ready-to-eat breakfast cereals, porridge, pasta and whole fruit. Future strategies to address the nutrient gaps identified among this population group could include the continued promotion of healthy food choices (including education around ‘healthy’ lifestyles and food marketing restrictions), improvements of the food supply through reformulation (fat, sugar, salt, dietary fibre), food fortification for micronutrients of concern (voluntary or mandatory) and/or nutritional supplement recommendations (for nutrients unlikely to be sufficient from food intake alone).

The childhood years represent a period of increased nutrient requirements during which a balanced diet is important to ensure optimal growth and development. It is well established that dietary habits and preferences are established early in life; thus, appropriate diet and lifestyle during this period can support optimal health and aid in the prevention of diet-related chronic diseases (e.g. obesity and CVD) in later life^(1–4)^. Regular surveillance of the dietary and lifestyle habits of children can help to inform targeted public health programmes to improve dietary patterns and adequacy during this critical period and future life stages.

Data from national dietary surveys globally have shown that high proportions of school-aged children (similar to other population groups) do not adhere to Food-Based Dietary Guidelines (FBDG) with low intakes of fruit, vegetables, wholegrains and dairy, and high intakes of animal proteins (particularly red and processed meat), discretionary foods and sugar-sweetened beverages (SSB) reported^(5–8)^. Furthermore, a recent review of nutrient intakes from national dietary surveys has highlighted high intakes of total fat, saturated fat, added sugars and salt and low intakes of carbohydrate and dietary fibre in school-aged children with large proportions of this population not meeting recommendations for vitamin D, folate and Fe^(9)^. Time-trend analyses have shown that over time (approx. 6–20 years), there have been reductions in intakes of fruit juice, meat (particularly red and processed meat), potatoes, milk (particularly whole milk) and SSB and increases in intakes of whole fruit and low-calorie drinks among children globally^(10–16)^. While these changes have resulted in decreased intakes of total and saturated fat, free sugars and Na and increased intakes of dietary fibre and some key micronutrients, for most nutrients, intakes are still not meeting recommendations among this population group^(10,15,17–19)^. While few national nutrition surveys have collected biomarkers of nutritional status among children, data from the UK National Diet and Nutrition Survey (NDNS) have reported low micronutrient status particularly with respect to vitamin D and folate among school-aged children^(20)^, while poor vitamin D status has also been reported in many population groups across Europe^(21)^.

In Ireland, the National Children’s Food Survey (NCFS) (2003–2004) found that school-aged children (5–12 years) had an energy-dense diet with high intakes of fruit juice, confectionery, SSB, saturated fat and Na and low intakes of fruit, vegetables, dietary fibre, vitamin A, vitamin D, folate, riboflavin, Ca and Fe^(22–24)^. Furthermore, studies of children in Ireland have reported low vitamin D and Fe status among children aged 2–14 years^(25,26)^. The current FBDG for the population aged over 5 years in Ireland were published in 2011 (translated into the visual representation of the Food Pyramid in 2016) and provide guidance on the consumption of six core food groups; ‘vegetables, salad and fruit’, ‘wholemeal cereals and breads, potatoes, pasta and rice’, ‘milk, yogurt and cheese’, ‘meat, poultry, fish, beans and nuts’, ‘fats, spreads and oils’ and the ‘top-shelf’ which includes ‘foods and drinks high in fat, sugar and salt’ (with only the first five food groups being recommended for good health)^(27,28)^. However, with a global emphasis to incorporate environmental sustainability and sociocultural factors in FBDG, it is necessary to have current information on dietary intakes of population groups to inform the development of revised guidelines^(29,30)^. Updated food consumption data have recently become available for school-aged children in Ireland through the National Children’s Food Survey II (NCFS II) (2017–2018) which utilised a similar methodology to the NCFS and offers a unique opportunity to determine the current food and nutrient intakes of this population group along with examining changes over time. Therefore, the aim of this study was to examine food and nutrient intakes and compliance with recommendations in school-aged children in Ireland from the NCFS II (2017–2018) and to examine changes since the previous NCFS (2003–2004).

## Experimental methods

### Study sample

Analyses were based on data from National Children’s Food Surveys in the Republic of Ireland (ROI), the NCFS (2003–2004) (*n* 594) and NCFS II (2017–2018) (*n* 600), two cross-sectional food consumption surveys conducted by the Irish Universities Nutrition Alliance (IUNA) (www.iuna.net) to establish databases of habitual food and beverage consumption in representative samples of school-aged children (5–12 years). Both studies were conducted according to the guidelines laid down in the Declaration of Helsinki, and ethical approval was obtained from St James’ Hospital and Federated Dublin Voluntary Hospitals Joint Research Ethics Committee for the NCFS and the Clinical Research Ethics Committee of the Cork Teaching Hospitals, University College Cork for the NCFS II. Written informed consent was obtained from children and their parents/guardians in both surveys.

### Sampling and recruitment methodology

Eligible participants were children aged between 5 and 12 years, inclusive. A total sample of 594 participants (boys: 293, girls: 301) (NCFS) and 600 participants (boys: 300, girls: 300) (NCFS II) were selected from databases of primary schools in the ROI provided by the Department of Education and Skills (previously Department of Education and Science during the NCFS). The databases were divided into: (i) small/medium/large schools; (ii) all boys/all girls/mixed; (iii) disadvantaged/not disadvantaged; and (iv) urban/rural. A random sample was selected so that, in the final sample, the proportions of children attending each of the categories reflected that of the proportions according to the database. The principals of selected schools were contacted with over 80 % of those contacted (in both surveys) agreeing to take part. Parents/guardians of children who were randomly selected from the school roll were contacted with information on the survey and participation was invited (one child per household only). Where families opted in, a researcher visited the home to explain the survey in more detail and to obtain written consent from children and their parents/guardians. Data collection was carried out from March 2003 to March 2004 for the NCFS and April 2017 to May 2018 for the NCFS II, providing a seasonal balance for both surveys. The overall response rates were 63 % for the NCFS and 65 % for the NCFS II. Demographic analysis of both survey samples demonstrated that they were nationally representative of children in Ireland with respect to age group, sex and geographical location when compared with the most recent Census data at that time^(31,32)^. While the NCFS was representative with respect to social class, the NCFS II contained a higher proportion of children of professional workers and a lower proportion of children of semi-skilled and unskilled workers than the national population. Consequently, all data presented in this manuscript with respect to the NCFS II were weighted to account for these differences.

### Food intake assessment

Food and beverage intake data (including nutritional supplements) were collected at brand level using a 7-d (NCFS) and 4-d (NCFS II) weighed food record (including at least one weekend day for all participants). The researcher made a number of visits to the participant and parents/guardians during the recording period: an initial training visit to demonstrate how to complete the food record and use the portable scales provided (Soehnle Vita 8020, London (NCFS) Tanita KD-400, Japan (NCFS II)), a second (and third for the NCFS) visit during the recording period to review the food record, check for completeness and clarify details regarding food descriptors and quantities, and a final visit 1–2 d after the recording period to review the final days of recording and to collect the food record. In both surveys, participants were asked to collect and provide the packaging labels for all foods, beverages and nutritional supplements consumed by the child over the recording period, to facilitate quantification and coding of foods.

### Food quantification

For both surveys, the majority of foods and beverages were weighed by the participant directly on the portable scales (NCFS: 75 %, NCFS II: 76 %) and a further 11 % of weights (for both surveys) were derived from manufacturer’s information on product labels. The remaining foods and beverages were quantified using photographic food atlases (NCFS: 5 %, NCFS II: 7 %)^(33,34)^, standard portion sizes (NCFS: 4 %, NCFS II: 3 %)^(35,36)^, household measures, (1 % for both surveys) and estimates based on the child’s previous eating patterns (used only when no other quantification method was appropriate) (NCFS: 3 %, NCFS II: 2 %). For all methods of quantification, leftovers were accounted for, and the weight of the food consumed was calculated.

### Estimation of food intakes

Each food, beverage and nutritional supplement consumed in both surveys was assigned a unique food code (at brand level) based on its descriptor and nutritional profile which was used to enter data into nutrition analysis software packages: WISP^©^ (Tinuviel Software, Anglesey, UK) (NCFS) and Nutritics^©^ (Nutritics, Dublin, Ireland) (NCFS II). Each food code was then categorised into food groups and further subdivided into smaller food groups guided by the FBDG for healthy eating in Ireland, for example, fruit and vegetables, breads (white/wholemeal), cereals (low/high fibre), potatoes (fresh/processed), milks (whole/reduced fat/non-dairy alternative), meats (fresh/processed), beverages (water/milk/soft drinks with added sugar/no added sugar soft drinks) and ‘top-shelf foods/foods high in fat, salt and sugar e.g. confectionery’. The mean daily intake (MDI) of each food group was calculated by summing the total intake per person over the recording period and dividing by the total number of recording days (NCFS: 7 d, NCFS II: 4 d). Consumers were defined as those who consumed at least one food or beverage belonging to the food group during the recording period.

**Table 1.** Distribution of food group intakes (g/d) in school-aged children (5–12 years) in Ireland, for the total population and percentage of consumers in the NCFS II and NCFS and the change (%) in intake (g/d) and consumers (%) between the NCFS and NCFS II (Mean values and standard deviations; median values and percentiles)

	NCFS (2003–2004) (*n* 594)						NCFS II (2017–2018) (*n* 600)						Change (%)	
	Mean	sd	Median	Percentiles		% Consumers	Mean	sd	Median	Percentiles		% Consumers	Mean (g/d)	% Consumers
				5th	95th					5th	95th			
Food groups	g/d						g/d							
Breads	82	39	76	29	153	100	85	45	78	22	179	99	+4·2	–1
*of which*														
White bread	63	37	57	11	128	98	53	42	48	0	128	89	–15·3*	–9*
Wholemeal and brown bread	12	24	0	0	61	40	25	36	9	0	97	54	+110*	+36*
Other breads	7	13	0	0	36	38	7	17	0	0	42	27	–1·4	–29*
Breakfast cereals	42	40	32	0	118	94	53	53	38	0	170	91	+26·8*	–3
*of which*														
Ready-to-eat breakfast cereals	31	24	27	0	81	93	28	23	25	0	73	85	–8·5	–8*
High-fibre cereals (≥ 6 g/100 g)	11	17	3	0	42	54	16	20	9	0	60	59	+44·2*	+9
Low-fibre cereals (< 6 g/100 g)	20	21	15	0	61	79	13	17	5	0	48	54	–37·1*	–31*
Porridge and hot oats cereals (made up)	11	36	0	0	84	17	25	51	0	0	148	28	+122*	+62*
Pasta, rice and savouries	58	50	47	0	152	89	72	61	57	0	197	89	+24·6*	0
*of which*														
Pasta	15	24	0	0	63	46	25	35	11	0	93	53	+63·7*	+16
Rice	10	19	0	0	46	36	12	23	0	0	57	32	+18·2	–12
Pizza	15	23	4	0	60	51	19	33	0	0	88	37	+27·5	–27*
Other cereals and savouries	18	29	0	0	78	50	16	33	0	0	80	38	–8·2	–23*
Potatoes and potato products	98	57	88	26	200	100	61	49	51	0	152	92	–38·0*	–8*
*of which*														
Boiled, baked and mashed potatoes	53	49	41	0	144	88	32	36	23	0	107	65	–40·1*	–26*
Chipped, fried and roasted potatoes	40	32	33	0	101	89	23	29	16	0	79	65	–41·8*	–27*
Processed and homemade potato products	6	15	0	0	26	28	6	20	0	0	38	18	+8·4	–35*
Milks	258	185	224	11	615	96	186	156	155	0	479	91	–28·0*	–6*
*of which*														
Whole milk	232	186	203	0	590	90	131	154	91	0	430	68	–43·3*	–25*
Reduced fat milk	26	86	0	0	174	17	52	110	0	0	298	31	+99·1*	+79*
Non-dairy products alternatives	0·2	4	0	0	0	0·5	3	19	0	0	0	3	+1115	+494
Yogurts and fromage frais	39	43	26	0	128	71	34	43	21	0	120	59	–12·2	–17*
Cheeses	8	10	4	0	28	59	11	13	6	0	38	63	+38·9*	+6
Creams, ice creams and dairy desserts	18	23	11	0	65	69	13	27	0	0	62	45	–27·1*	–35*
Butter and spreading fats	9	8	8	0	21	93	7	7	5	0	19	87	–23·9*	–7*
Meat and meat dishes	105	55	102	30	202	98	116	69	105	26	252	98	+10·5	0
*of which*														
Meat dishes	37	40	26	0	114	71	47	58	30	0	158	64	+28·5*	–10
Processed meat	46	30	41	3	104	95	41	36	34	0	105	91	–9·9	–5
Fresh meat	23	22	16	0	64	86	28	31	20	0	85	73	+22·5	–15*
Fish and fish dishes	9	14	0	0	34	48	13	29	0	0	56	41	+47·3	–15
*of which*														
White/oily/other fish	6	9	0	0	26	40	10	25	0	0	42	37	+77·2*	–7
Fish dishes	1	8	0	0	0	4	3	14	0	0	15	7	+103	+59
Eggs and egg dishes	8	13	0	0	36	42	10	17	0	0	47	35	+23·9	–17
Nuts and seeds	0·4	2	0	0	4	8	0·7	4	0	0	5	8	+64·3	–1
Fruit and vegetables	224	153	186	35	525	100	221	129	201	53	444	100	–1·0	0
Fruit and fruit juices	155	133	123	7	427	98	147	115	125	10	349	97	–4·8	–1
*of which*														
Discrete fruit	59	53	46	0	161	85	90	70	78	0	229	92	+53·1*	+8*
Fruit in composite dishes	9	11	6	0	28	94	9	14	5	0	31	84	–2·8	–10*
Fruit juice (100 % fruit)	86	113	46	0	314	67	38	75	0	0	161	40	–56·3*	–40*
Smoothies	0·6	6	0	0	0	2	11	36	0	0	90	12	+1715*	606*
Vegetables	69	52	58	6	165	98	74	49	67	12	165	99	+7·4	+1
*of which*														
Discrete vegetables	39	35	31	0	104	92	40	38	30	0	111	85	+4·1	–7
Vegetables in composite dishes	30	28	23	1	83	97	34	32	25	0	96	95	+11·5	–2
Confectionery	85	42	79	28	161	100	76	48	67	17	162	99	–10·1	–1
Water as a beverage	–	–	–	–	–	–	450	315	405	22	1037	95		
Soft drinks†	331	285	276	18	821	95	160	221	83	0	595	67	–51·7*	–29*
*of which*														
Soft drinks, no added sugar	78	206	0	0	433	40	110	201	0	0	483	47	+40·3	+16
Soft drinks, added sugar	252	227	194	0	677	93	50	93	0	0	217	40	–80·2*	–57*
Milk as a beverage	–	–	–	–	–	–	91	127	47	0	357	58		
Teas and coffees	36	72	0	0	179	42	29	81	0	0	203	19	–21·4	–55*
Sweetened milk drinks	15	36	0	0	79	23	16	43	0	0	89	19	+8·8	–17

* Denotes statistical differences (*P* < 0·001) between the NCFS and NCFS II via independent-samples *t* test and adjusted for multiple testing.

† Carbonated beverages, fruit juice drinks, squashes and cordials.

### Estimation of nutrient intakes

Nutrient intakes were estimated using WISP^©^ for the NCFS and Nutritics^©^ for the NCFS II, both of which use food composition data from McCance and Widdowson’s ‘*The Composition of Foods’*; 6th Ed, 5th Ed and all nine supplemental volumes (NCFS) and the 7th Ed (and 6th Ed for a small number of foods) (NCFS II)^(37–39)^. During both surveys, modifications were made to include recipes of composite dishes, nutritional supplements, fortified foods and generic Irish foods that were commonly consumed. As ‘*The Composition of Foods’* does not contain values for some potentially important sources of dietary fibre (e.g. some fruits, vegetables and cereal products) and vitamin D (e.g. white fish, salmon, processed meat (including ham), mushrooms and milk), values were updated using data from published food composition databases as appropriate^(40–43)^ and as some nutrient values differ greatly by brand, food packaging labels were used to update values for dietary fibre and Na. Furthermore, the fatty acid composition has been updated and is outlined elsewhere^(44)^. Free sugar values were assigned by adapting a systematic approach used to calculate added sugars content in foods and beverages^(45)^ and guidance from Public Health England on the calculation of free sugars^(46)^. The folic acid composition of fortified foods and nutritional supplements was established from the food packaging labels or obtained directly from the manufacturer. Dietary folate equivalents (DFE) were estimated as 1 µg DFE = 1 µg food folate + (1·7 × folic acid)^(47)^.

### Estimation of usual nutrient intakes

Usual intake distributions for energy and nutrients were estimated using the validated National Cancer Institute (NCI) method^(48)^ which accounts for both inter- and intra-person variance. The NCI method has been implemented in SAS macros (version 2.1) which were downloaded from www.riskfactor.cancer.gov/diet/usualintakes/macro.html (date of download: July 2015). For these analyses, the covariates used were sex and age group (5–8 years and 9–12 years).

### Estimation of sodium intake from spot urine sample

For the NCFS II, Na intakes were calculated by correcting the population mean Na values from spot urine samples for sex- and age-specific 24-h urine output volumes derived from a study on Australian children (in the absence of data for children in Ireland)^(49)^. Participants (95 % of sample; *n* 572) provided a first void morning urine sample (about 30 ml) during the recording period. The sample was then sealed in the sterile container and stored in cool conditions (wrapped in an ice pack and kept in a thermal cooler bag) prior to same day collection by the researcher. Once collected, the urine sample was stored on dry ice by the researcher and transported to the university laboratories for storage at –20°C, until processing. Urinary Na was measured by Randox Laboratories using a Randox Rx Daytona with an ion-selective electrode. All samples were analysed in duplicate, and the average of the two readings was calculated. Interassay coefficient for Na was < 2·2 %.

### Comparison of energy and nutrient intakes with dietary reference values

Nutrient intakes were compared with the most recent Dietary Reference Values (DRV) available from the European Food Safety Authority (EFSA). The UK Department of Health (DoH) or Scientific Committee on Nutrition (SACN) DRV were used if they were not superseded by updated DRV from EFSA. Mean protein intake (g/kg body weight) was compared with the age- and sex-specific average requirement (AR) and population reference intake set by EFSA^(47)^. Mean intakes for carbohydrate and fat were compared with the average population intake recommended by the UK SACN for carbohydrate (50 % Energy (%E))^(46)^ and the UK DoH for total fat (< 35 %E)^(50)^. Mean intakes of saturated fat, MUFA and PUFA were compared with the UK DoH recommendations for a population mean intake < 10 %E for saturated fat and the minimum average population intake recommendations of 12 %E for MUFA and 6 %E for PUFA^(50)^. Mean dietary fibre intakes were compared with the adequate intake (AI) from EFSA (4–6 years: 14 g/d, 7–10 years: 16 g/d and 11–14 years: 19 g/d)^(47)^. Mean intake of free sugars was compared with the WHO recommendation of < 10 %E for individuals and the UK SACN recommendation for an average population intake < 5 %E^(46,51)^. Urinary salt excretion was compared with the maximum population targets set by the Food Safety Authority of Ireland (FSAI) (5–6 years: 3 g/d, 7–10 years: 5 g/d and 11–12 years: 6 g/d)^(52)^.

### Adequacy of micronutrient intakes

The prevalence of inadequate intakes of micronutrients was estimated using estimated average requirements (EAR) as cut points. This method has been shown to be effective in obtaining a realistic estimate of the prevalence of dietary inadequacy^(53)^. EAR established by the EFSA were used as cut-offs for assessing the prevalence of inadequate intakes of vitamin A, thiamin, riboflavin, total niacin equivalents, vitamin B_6_, DFE, vitamin C, Ca, Fe and Zn^(47)^. EAR established by the UK DoH were used for assessing the prevalence of inadequate intakes of thiamin, vitamin B_12_ and Mg^(50)^. The US Insitiute of Medicine (IOM) EAR of 10 µg/d and the Nordic EAR of 7·5 µg/d were used to assess the prevalence of inadequate vitamin D intakes^(54,55)^. As under-reporting of food consumption can result in an overestimate of the prevalence of inadequacy in a population group^(56)^, under-reporters (UR) were identified and excluded from these analyses (NCFS: 32·5 % of total sample, NCFS II: 19·5 %). Under-reporters were identified in the NCFS as previously outlined^(57)^ and in the NCFS II using Goldberg’s cut-off2 criterion updated by Black (which evaluates the ratio of energy intake to BMR against age-specific energy cut-offs based on physical activity levels)^(47,58–60)^.

### Risk of excessive intakes of micronutrients

The risk of excessive intake of micronutrients was evaluated using the tolerable upper intake level (UL) as a reference value. The UL is defined as the maximum level of total chronic daily intake of a nutrient (from all sources) judged to be unlikely to pose a risk of adverse health effects to humans^(61)^. UL established by the EFSA/EU Scientific Committee for Food were used for vitamin A (retinol), vitamin D, vitamin E, pre-formed niacin, vitamin B_6_, folic acid, Ca, Mg and Zn^(61–63)^. UL established by the US Food and Nutrition Board (FNB) were used for Fe and vitamin C^(64,65)^.

### Percentage contribution of food groups to energy and nutrient intakes

The percentage contribution of food groups to intakes of energy and nutrients from the NCFS II were calculated by the mean proportion method^(66)^ using SPSS^©^ for Windows™ version 26.0 and are presented in Supplementary Tables 1–3. This method provides information about the sources that are contributing to the nutrient intake ‘per person’ and is the preferred method when determining important food sources of a nutrient for individuals in the population group as opposed to investigating the sources of a nutrient within the food supply.

### Statistical analysis

Statistical analyses were carried out using SPSS^©^ for Windows™ version 26.0. Changes between the two surveys were calculated by:

((NCFS II value – NCFS value)/NCFS value) × 100 (in %)

Differences in intakes between the NCFS (2003–2004) and NCFS II (2017–2018) were assessed using independent-samples *t* tests regardless of normality (due to the large sample size) for continuous variables and *χ* ^2^ tests for categorical variables. As sample size increases so does the robustness of *t* tests to identify deviations from normality, thus parametric tests are recommended for large samples^(67)^. To minimise type 1 errors (as a result of multiple testing), the Bonferoni adjustment was used by dividing the α level (0·05) by the number of comparisons. Therefore, intakes were considered to be significantly different from each other if *P* < 0·001. However, due to the large sample in this study even a small difference between group means was highly statistically significant; thus, greater emphasis was placed on a descriptive, rather than a formal statistical analysis of the data.

## Results

Table 1 presents the percentage of consumers and the MDI of food groups in school-aged children (5–12 years) in the NCFS (2003–2004) and the NCFS II (2017–2018) and the changes in food group intakes between the two surveys. In the NCFS II, ‘breads’ were consumed by 99 % of children with a MDI of 85 g (white bread 53 g, wholemeal and brown bread 25 g, and other breads 7 g). The MDI of ‘breads’ is unchanged since the NCFS (2003–2004) (82 g); however, there has been a decrease in the MDI of white bread (63 *v*. 53 g) and an increase in the MDI of wholemeal and brown bread (12 *v*. 25 g). ‘Breakfast cereals’ were consumed by 91 % of children in the NCFS II with 85 % consuming ready-to-eat breakfast cereals (RTEBC) and 28 % consuming ‘porridge and hot oat cereals’. The MDI of RTEBC was 28 g (high-fibre RTEBC: 16 g, low-fibre RTEBC: 13 g), and the MDI of ‘porridge and hot oat cereals’ was 25 g. Since the NCFS (2003–2004), there has been an increase in the MDI of high-fibre RTEBC (11 *v*. 16 g) and ‘porridge and hot oat cereals’ (11 *v*. 25 g) and a decrease in the MDI of low-fibre RTEBC (20 *v*. 13 g).

In the NCFS II, ‘pasta, rice and savouries’ were consumed by 89 % of children with a MDI of 72 g (pasta 25 g, rice 12 g, pizza 19 g, other cereals and savouries 16 g). Overall, the MDI of ‘pasta, rice and savouries’ has increased since the NCFS (2003–2004) (58 *v*. 72 g) attributable to an increase in the MDI of pasta (15 *v*. 25 g). In the NCFS II, ‘potatoes and potato products’ were consumed by 92 % of children with a MDI of 61 g (boiled, baked and mashed potatoes 32g; chipped, fried and roasted potatoes 23g; and processed and homemade potato products 6 g). Since the NCFS (2003–2004), there has been a decrease in the MDI of ‘potatoes and potato products’ (98 *v*. 61 g) attributable to a decrease in the MDI of boiled, baked and mashed potatoes (53 *v*. 32 g) and chipped, fried and roasted potatoes (40 *v*. 23 g).

In the NCFS II, ‘milks’ were consumed by 91 % of children with a MDI of 186 g (whole milk 131 g, reduced-fat milk 52 g and non-dairy alternatives 3 g). Approximately two-thirds of children consumed yogurts and fromage frais (59 %) and cheese (63 %) with a MDI of 34 and 11 g, respectively. Since the NCFS (2003–2004), there has been a decrease in the MDI of total milk (258 *v*. 186 g) and whole milk (232 *v*. 131 g) and an increase in the MDI of reduced fat milk (26 *v*. 52 g). The MDI of cheese has also increased (8 *v*. 11 g), while there has been a decrease in the MDI of ‘creams, ice-creams and dairy desserts’ (18 *v*. 13 g) and ‘butter and spreading fats’ (9 *v*. 7 g).

In the NCFS II, ‘meat and dishes’ were consumed by 98 % of children with a MDI of 116 g (meat dishes 47 g (of which fresh meat dishes 44 g), processed meat 41 g and fresh meat 28 g). Overall, the MDI of total meat, processed meat and fresh meat was unchanged since the NCFS (2003–2004); however, the MDI of meat dishes has increased (37 *v*. 47 g). In the NCFS II, ‘fish and fish dishes’ were consumed by 41 % of children with a MDI of 13 g (discrete fish, e.g. white/oily/other 10 g, fish dishes 3 g). Overall, the MDI of total fish is unchanged since the NCFS (2003–2004); however, there has been an increase in the MDI of discrete fish (6 *v*. 10 g). In the NCFS II, 35 % of children consumed ‘eggs and egg dishes’ with a MDI of 10 g and 8 % consumed ‘nuts and seeds’ with a MDI of < 1 g, both of which were similar to the previous NCFS (2003–2004).

In the NCFS II, ‘fruit and vegetables’ were consumed by all children (100 %) with a MDI of 221 g (discrete fruit 90 g, fruit juice 38 g, smoothies 11 g, fruit in composite dishes 9 g, discrete vegetables 40 g and vegetables in composite dishes 34 g). Overall, the MDI of total fruit and vegetables is unchanged since the NCFS (2003–2004) (224 g); however, there has been an increase in the MDI of discrete fruit (59 *v*. 90 g) and smoothies (< 1 *v*. 11 g) and a decrease in the MDI of fruit juice (86 *v*. 38 g). The proportion of consumers and the MDI of total vegetables, discrete vegetables and vegetables from composite dishes is unchanged since the NCFS (2003–2004).

In the NCFS II, confectionery products (biscuits, cakes, confectionery and savoury snacks) were consumed by 99 % of children with a MDI of 76 g, similar to that in the NCFS (2003–2004) (100 % consumers and MDI of 85 g). With regard to beverage intakes, water was the most commonly consumed beverage in the NCFS II (95 % consumers) with a MDI of 450 g. Milk was consumed as a beverage by 58 % of children, with a MDI of 91 g. Soft drinks were consumed by 67 % of children with a MDI of 160 g (no added sugar variety 110 g and sugar-sweetened 50 g). Since the NCFS (2003–2004), there has been a decrease in the MDI of total soft drinks (331 *v*. 160 g) and sugar-sweetened soft drinks (252 *v*. 50 g), while the MDI of no added sugar soft drinks is unchanged. ‘Teas and coffees’ and sweetened milk drinks were consumed by 19 % of children in the NCFS II with a MDI of 29 and 16 g, respectively, which was similar to the previous NCFS (2003–2004).

Table 2 presents the mean intake of energy and nutrients in school-aged children (5–12 years) in Ireland in the NCFS (2003–2004) and the NCFS II (2017–2018) and the changes in intakes between the two surveys. The percentage contribution of food groups to energy and nutrient intakes (key sources) in the NCFS II are presented in Supplementary Tables 1–3. In the NCFS II, the mean intake of energy was 6·3MJ with a decrease of approximately 200 kcal since the NCFS (2003–2004) (7·0MJ). The mean intake of protein in the NCFS II was 2·0 g/kg body weight per d which is equivalent to over two times the population reference intake, and no child had intakes below the AR (indicating that protein intakes are adequate among this population group). The mean intake of protein has increased since the previous NCFS (1·8 *v*. 2·0 g/kg body weight). The mean intake of fat in the NCFS II was 33 %E which is below the recommendation of < 35 %E and is similar to that in the NCFS (34 %E). The mean intake of saturated fat in the NCFS II was 14 %E and despite a decrease since the previous NCFS (15 %E), it is still above the recommendation of < 10 %E. The mean intakes of MUFA (14 %E) and PUFA (6 %E) are in line with recommendations (MUFA ≥ 12 %E, PUFA ≥ 6 %E) and have increased since the NCFS (MUFA 12 %E, PUFA 5 %E). The mean intake of carbohydrate in the NCFS II was 50 %E which meets the recommendation for an average population intake of 50 %E but has decreased since the previous NCFS (52 %E). The mean intake of free sugars in the NCFS II was 9 %E which is above the UK SACN recommendation for an average population intake < 5 %E, with 40 % of children having intakes above the WHO recommendation of < 10 %E; however, there has been a notable decrease in the intake of free sugars since the NCFS (16 %E). The mean intake of dietary fibre in the NCFS II was 15 g and while intake has increased since the NCFS (12 g), it is still below the AI for older children (≥ 7 years). There has been an increase in the mean intakes of vitamin D, vitamin E and Zn since the NCFS (2003–2004), while mean intakes of thiamin, riboflavin, vitamin B_6_, total folate, DFE, vitamin C, potassium, Ca and Cu have decreased. The mean intake of salt (calculated as Na equivalents) from food sources only (excluding discretionary salt) was 4 g which has decreased since the NCFS (5 g). However, the MDI of Na from all sources (calculated from urinary output) was 5 g and was above the FSAI maximum population targets for older children (≥ 7 years).

**Table 2.** Distribution of energy, macronutrients, dietary fibre, vitamin and mineral intakes in school-aged children (5–12 years) in Ireland in the NCFS II and NCFS and the change in intakes between the NCFS and NCFS II (Mean values and standard deviations)

	NCFS (2003–04) (*n* 594)					NCFS II (2017–18) (*n* 600)					
				Percentiles					Percentiles		Change
	Mean	sd	Median	5th	95th	Mean	sd	Median	5th	95th	Mean (%)
Macronutrients and fibre											
Energy (MJ)	7·0	1·4	7·0	4·9	9·4	6·3	1·2	6·2	4·5	8·3	–10·8*
Energy (kcal)	1667	323	1653	1166	2229	1487	275	1469	1067	1969	–10·8*
Protein (g)	56·6	13·4	55·8	36·4	80·5	59·6	14·1	58·0	39·2	85·3	+5·2*
Protein (g/kg body weight)	1·8	0·7	1·7	0·8	3·1	2·0	0·6	2·0	1·1	3·1	11·7*
%E from protein	13·7	1·8	13·7	10·9	16·9	16·2	2·1	16·0	13·0	19·9	+17·7*
Total fat (g)	63·1	14·2	62·2	41·4	88·1	55·8	12·7	54·8	36·8	78·5	–11·5*
%E from total fat	33·7	3·6	33·7	27·8	39·6	33·3	3·8	33·3	27·0	39·6	–1·2
Saturated fat (g)	27·3	6·7	26·8	17·1	39·1	23·4	5·6	23·0	15·0	33·4	–14·1*
%E from saturated fat	14·5	2·1	14·5	11·0	18·1	14·0	2·2	14·0	10·6	17·7	–3·5*
Monounsaturated fat (g)	21·5	5·1	21·2	13·9	30·5	22·9	5·6	22·4	14·7	32·9	+6·2*
%E from MUFA	11·5	1·5	11·5	9·1	14·0	13·6	1·8	13·6	10·7	16·6	+18·1*
Polyunsaturated fat (g)	9·2	2·8	8·9	5·3	14·2	9·3	2·5	9·0	5·8	13·8	+1·2
%E from PUFA	4·9	1·0	4·8	3·4	6·7	5·5	1·0	5·5	4·1	7·3	+13·4*
Carbohydrate (g)	231	47	228	158	313	197	38·3	195	138	264	–14·6*
%E from carbohydrate	52·1	4·2	52·1	45·2	59·0	50·0	4·3	50·1	42·8	57·0	–4·0*
Total sugars (g)	107	29·3	104	62·9	159	73·7	19·4	72·3	44·4	108	–30·8*
%E from total sugars	23·9	4·7	23·7	16·5	32·1	18·7	4·0	18·5	12·5	25·6	–21·7*
Free sugars (g)	73·4	26·2	70·7	35·7	121	38·4	14·9	36·6	17·4	65·6	–47·7*
%E from free sugars	16·3	5·0	16·0	8·8	25·2	9·5	3·4	9·2	4·5	15·6	–41·9*
Dietary fibre (g)	12·4	3·5	12·1	7·3	18·8	15·1	3·6	14·8	9·8	21·6	+21·9*
Dietary fibre (g/10MJ)	17·9	3·7	17·7	12·3	24·4	24·7	4·5	24·4	17·9	32·6	+37·8*
Vitamins†											
Total vitamin A (µg)	700	414	613	217	1507	666	307	613	274	1243	–5·0
Retinol (µg)	347	237	293	77	811	292	146	268	101	565	–15·8*
Carotene (µg)	2130	1688	1683	496	5368	2397	1828	1920	600	5823	+12·5*
Vitamin D (µg)	2·5	1·8	2·1	0·5	6·0	4·2	3·1	3·5	0·9	10·1	+68·5*
Vitamin E (mg)	6·3	3·2	5·8	2·2	12·3	6·9	2·9	6·5	3·0	12·4	+9·9*
Thiamin (mg)	1·5	0·5	1·4	0·8	2·5	1·4	0·4	1·4	0·8	2·2	–6·9*
Riboflavin (mg)	1·8	0·8	1·7	0·8	3·3	1·6	0·6	1·5	0·8	2·6	–13·4*
Total Niacin (mg)	28·5	7·8	27·8	17·0	42·5	28·8	7·5	28·0	18·2	42·5	+1·3
Vitamin B_6_ (mg)	2·0	0·7	1·9	1·0	3·4	1·5	0·5	1·4	0·8	2·5	–24·7*
Vitamin B_12_ (µg)	4·4	1·9	4·2	1·8	7·9	4·6	1·8	4·4	2·1	8·0	+5·1
Total folate (µg)	224	82	214	108	377	211	65	204	117	330	–6·0*
DFE (µg)	268	109	253	119	473	253	97	238	122	432	–5·6*
Biotin (µg)	24·8	12·9	22·2	9·3	49·8	25·3	11·2	23·3	10·9	46·4	+2·0
Pantothenate (mg)	5·3	2·0	5·1	2·6	9·1	5·3	1·8	5·1	2·8	8·8	+0·4
Vitamin C (mg)	86	50	76	25	182	73	42	65	22	154	–15·0*
Minerals†											
Na (mg)	2081	506	2047	1317	2981	1657	365	1627	1114	2310	–20·4*
Potassium (mg)	2190	502	2164	1418	3073	2019	486	1980	1296	2891	–7·8*
Ca (mg)	862	264	841	470	1337	791	241	767	438	1224	–8·2*
Fe (mg)	9·4	3·0	9·0	5·1	14·8	9·0	2·4	8·7	5·4	13·4	–4·2
Mg (mg)	194	47	191	122	277	194	47	190	124	279	+0·3
Zn (mg)	6·6	1·8	6·4	4·0	10·0	7·8	2·1	7·5	4·8	11·6	+17·6*
Cu (mg)	0·8	0·3	0·8	0·4	1·3	0·8	0·2	0·7	0·5	1·1	–5·6*
Phosphorus (mg)	1024	258	1008	630	1481	1008	256	986	628	1467	–1·5

DFE, dietary folate equivalents.

* Statistical differences (*P* < 0·001) between the NCFS and NCFS II via independent-samples *t* test and adjusted for multiple testing.

† All sources including nutritional supplements.

Table 3 presents the proportion of school-aged children (5–12 years) in Ireland with micronutrient intakes below the EAR in the NCFS (2003–2004) and the NCFS II (2017–2018). In the NCFS II, a significant proportion of children had inadequate intakes of vitamin D (94 %), Ca (37 %), Zn (24 %), Fe (20 %), vitamin C (19 %), Mg (18 %) and DFE (13 %). The proportion of children with inadequate intakes of vitamin D and Zn has decreased since the previous NCFS (2003–2004), while the proportion with inadequate intakes of vitamin B_6_ and Ca has increased. The proportion of children with intakes exceeding the UL for retinol, vitamins D, E, C, B_6_, preformed niacin, folic acid, Ca and Fe was < 0·5 % in both surveys. In the NCFS II, a very small proportion of children (2 %) had Zn intakes exceeding the UL (< 1 % in the NCFS (2003–2004)).

**Table 3.** Proportion of school-aged children (5–12 years) in Ireland with micronutrient intakes below the estimated average requirement (EAR) (excluding under-reporters) in the NCFS and NCFS II

		% < EAR			
		NCFS		NCFS II	
	Estimated average requirement	(2003–04)		(2017–18)	
Vitamin A	245 µg/d (4–6 years)	13		8·9	
	320 µg/d (7–10 years)^(47)^				
	480 µg/d (11–14 years)^(47)^				
Vitamin D	10 µg/d^(54)^	100		94*	
	7·5 µg/d^(55)^	100		86*	
Thiamin	0·072 mg/MJ^(47)^	0		0	
Riboflavin	0·6 mg/d (4–6 years)^(47)^	3		5	
	0·8 mg/d (7–10 years)^(47)^				
	1·1 mg/d (11–14 years)^(47)^				
Total niacin equivalents	1·3 mg NE/MJ^(47)^	0		0	
Vitamin B_6_	0·6 mg/d (4–6 years)^(47)^	2		8*	
	0·9 mg/d (7–10 years)^(47)^				
	1·2 mg/d (11–14 years)^(47)^				
Vitamin B_12_	0·7 µg/d (4–6 years)^(50)^	0		0	
	0·8 µg/d (7–10 years)^(50)^				
	1·0 µg/d (11–14 years)^(50)^				
Dietary folate equivalents	110 µg/d (4–6 years)^(47)^	11		13	
	160 µg/d (7–10 years)^(47)^				
	210 µg/d (11–14 years)^(47)^				
Vitamin C	25 mg/d (4–6 years)^(47)^	13		19	
	40 mg/d (7–10 years)^(47)^				
	60 mg/d (11–14 years)^(47)^				
Ca	680 mg/d (4–10 years)^(47)^	24		37*	
	960 mg/d (11–17 years)^(47)^				
Fe	5 mg/d (1–6 years)^(47)^	20		20	
	8 mg/d (7–11 years)^(47)^				
	8 mg/d (boys, 12 years)^(47)^				
	7 mg/d (girls, 12 years)^(47)^				
Mg	90 mg/d (4–6 years)^(50)^	18		18	
	150 mg/d (7–10 years)^(50)^				
	230 mg/d (11–14 years)^(50)^				
Zn	4·6 mg/d (4–6 years)^(47)^	36		24*	
	6·2 mg/d (7–10 years)^(47)^				
	8·9 mg/d (11–14 years)^(47)^				

* Statistical differences (*P* < 0·001) between the NCFS and NCFS II via *χ* ^2^ test and adjusted for multiple testing.

## Discussion

This study provides information on the current food and nutrient intakes and compliance with recommendations in school-aged children (5–12 years) in Ireland and is the first study to examine changes over time among this population group. This study has found that while overall intakes of macronutrients (protein, fat and carbohydrate) are generally sufficient for this population group, intakes of saturated fat, free sugars and salt are higher, and intakes of dietary fibre are lower than recommendations. Furthermore, a high proportion of this population group have inadequate intakes of key micronutrients including vitamin D, Ca, Fe and folate. Some of the key dietary changes that have occurred since the NCFS (2003–2004) include lower intakes of SSB, fruit juice, milk and potatoes, and higher intakes of wholemeal/brown bread, high-fibre RTEBC, porridge, pasta and whole fruit. While these changes have resulted in some improvements in nutrient intakes (i.e. decreased saturated fat, free sugars and salt, and increased dietary fibre and some key micronutrients), gaps remain with respect to recommendations for a number of nutrients which are discussed below in the context of the literature and public health implications. As dietary patterns are mainly developed at a young age and can track into adolescence and adulthood, it is important to address these intakes in this population group to reduce the risk of development of chronic diseases such as overweight/obesity, hypertension and CVD throughout the life cycle^(1–4,68)^.

This study found that the mean intake of saturated fat among school-aged children in Ireland in 2017–2018 (14 %E) has decreased since 2003–2004 (15 %E) but still exceeds recommendations (< 10 %E), which is consistent with findings from other national dietary surveys of children where intakes of saturated fat have also decreased over time but are still too high^(10,15,17)^. The decrease of saturated fat in this study may be partly explained by the reduction in milk intake between 2017–2018 and 2003–2004 as the intake of other key sources of saturated fat (meats, biscuits and confectionery) were unchanged (online Supplementary Table 1). A decrease in milk intake has also been observed in other studies of children but in contrast to our findings, the intake of meat (particularly processed meat) has also decreased^(10–16)^. There is currently a growing body of evidence suggesting that the dairy food matrix (in addition to providing key nutrients such as Ca and iodine) may be beneficial in terms of cardiovascular health^(69)^, and that the consumption of ultra-processed foods (e.g. SSB, confectionery products and processed meats) is associated with poor nutritional quality and an increased risk of negative health outcomes in populations^(70)^. Therefore, public health efforts to reduce saturated fat intakes for health should focus on reducing the consumption of low-nutrient-dense foods such as processed meat and ‘top-shelf’ foods including confectionery.

Despite a notable decrease in free sugars intake observed in this study between 2017–2018 and 2003–2004 (9 *v*. 16 %E), intakes are still above the SACN recommendation for a maximum population intake of < 5 %E and 40 % of school-aged children in Ireland have intakes above the WHO recommendation of < 10 %E^(46,51)^. Data from the UK and Germany have also shown that despite reductions in free sugar intakes among children over time, intakes still exceed recommendations^(10,15)^. The decrease in free sugars intake noted in children in Ireland since 2003–2004 can be explained by the decreased intake of fruit juices and SSB (as intakes of other sources including biscuits, confectionery, breakfast cereals and yogurts were unchanged (online Supplementary Table 1)). Similarly, intakes of fruit juice and SSB have decreased over time among children in Australia, Canada, the UK and the USA^(11–13,15,16)^. While many countries have implemented strategies to reduce sugar intake including sugar tax policies, reformulation initiatives and education policies (with some successes), further research is needed to determine the long-term impact of these policies on actual sugar intakes and health outcomes of populations^(71)^.

Dietary fibre intake increased in school-aged children in Ireland between 2017–2018 and 2003–2004 (15 *v*. 12 g); however, intakes among children ≥ 7 years are still below the AI set by EFSA for normal bowel function^(47)^. Similarly, despite increases over time, dietary fibre intakes are still below recommendations for children in Germany, the UK and the USA^(10,15,18)^. The modest increase in dietary fibre intake since 2003–2004 may be explained by the increase in intake of wholemeal/brown bread, high-fibre RTEBC and whole fruit. Notably, the increased contributions to dietary fibre intake from high-fibre RTEBC are likely to be due to reformulation rather than active decisions/behaviour change as a direct comparison of brands between the two surveys in this study found that some were previously categorised as low fibre (< 6 g/100 g) in 2003–2004. Studies from Australia, Canada, Germany, the Netherlands and the USA have also shown increases in intakes of wholemeal/brown cereals and fruit among children over time^(10,11,13,14,16)^.

This study found that salt intakes in school-aged children in Ireland generally exceeded recommendations set by the FSAI. However, Na intakes (from food sources only) were found to have decreased by approximately 1 g since 2003–2004 which may be attributable to the FSAI’s salt reduction programme (implemented in 2003) which has resulted in significant reductions in the salt content of a number of foods, particularly breads and processed meats^(72)^ (key sources of salt among this population group (online Supplementary Table 3)). Similar salt reduction programmes have been implemented in other European countries which have also resulted in significant reductions in Na intakes in populations including children^(15,73)^. As high blood pressure in childhood has been shown to be associated with high blood pressure and subsequent CVD risk in later life, early intervention is crucial to ensure the development of appropriate dietary patterns for optimal lifelong health^(4)^. However, as this study has found that intakes of breads and processed meats are generally stable over time, further reformulation efforts in conjunction with dietary strategies will be needed if target salt recommendations are to be met.

With regard to micronutrient intakes, this study found that a significant proportion of school-aged children in the NCFS II (2017–2018) had inadequate intakes of vitamin D (94 %), Ca (37 %), Zn (24 %), Fe (20 %), vitamin C (19 %), Mg (18 %) and DFE (13 %) which are important for lifelong bone health, cognitive and behavioural development, and general immune health^(47,50,74)^. Adequate intakes of Ca, vitamin D and Mg are essential during childhood for lifelong bone health (peak bone mass developed in the first 25 years of life is an important determinant of osteoporosis in later life). While this study found that the intake of vitamin D in 2017–2018 has increased since 2003–2004 (4·2 *v*. 2·5 µg), almost all children (94 %) still have inadequate intakes (based on adequate serum 25(OH)D status deemed to be adequate for bone health)^(54)^. The increase in vitamin D intake may be partly explained by the increased contribution of vitamin D-fortified RTEBC in the NCFS II (0·93 µg/d) compared with the NCFS (0·24 µg/d). The intake of Ca in 2017–2018 has decreased since 2003–2004 (partly explained by the reduction in milk intake) and the MDI of dairy products is well below the recommended 3–5 servings/d for this age group (about 1·6 servings/d) resulting in 37 % of children having inadequate intakes of Ca. While a proportion of children in this study were also found to have low Mg intakes, this may be due to the DRV used as there is currently no clinical evidence of low Mg intakes in EU populations and EFSA have called for further research to support the evidence base for setting DRV for Mg^(75,76)^. Low intakes of vitamin D (along with insufficient biochemical status), Ca and Mg have also been reported in other dietary surveys of children with reductions in milk and dairy products also reported over time^(10–13,15,16,19,77)^. As there are few natural sources of vitamin D, food-based strategies (e.g. fortification) and nutritional supplement recommendations/policies may be necessary to meet requirements (particularly for populations at northerly latitudes or those with limited time outdoors)^(78)^. In Ireland, the FSAI recommend a 10 µg/d vitamin D supplement for all ages (particularly in winter), but this recommendation has not yet been implemented into policy for school-aged children and is not widely adhered to with just 19 % of children in the NCFS II taking a supplement containing vitamin D (with < 3 % taking > 10 µg/d)^(79)^.

This study found that 20 % of school-aged children in Ireland have inadequate intakes of Fe which is similar to findings from other countries where low Fe intakes have been noted in children^(10,15,80)^. Furthermore, data from the UK NDNS have shown that 6 % of boys and 13 % of girls (4–10 years) have low Fe stores with approximately 5 % showing evidence of anaemia^(20)^. Addressing the low intakes in this population group is necessary to promote cognitive and behavioural development and is of particular importance for older girls due to the onset of menstruation, which may elevate the risk of low Fe stores and Fe deficiency anaemia^(81)^. The mean intake of Fe was similar in 2017–2018 compared with 2003–2004 which may be explained by the relatively stable consumption of the key sources of Fe including fortified RTEBC, breads and meat (online Supplementary Table 3).

Folate intake (measured by DFE) decreased modestly between 2017–2018 and 2003–2004 with 13 % of school-aged children in Ireland in the NCFS II having inadequate intakes for normal cell development and growth. While data from other countries have shown similar or slightly increased folate intakes over time, intakes are still below recommendations in children^(10,15)^. Biochemical status data from the UK NDNS have reported that 4 % of children (4–10 years) had red blood cell folate below the threshold for folate deficiency and 17 % below the threshold for serum folate indicating possible deficiency^(20)^. Key sources of DFE in the NCFS II were folic acid-fortified RTEBC, breads, milks, fruit and vegetables (online Supplementary Table 2), and as intakes of milk, fruit and vegetables were below recommendations among children in Ireland, continued promotion of healthy food choices in line with FBDG guidelines (along with fortified foods) may help to improve intakes of folate in this population group.

The current study also found that a proportion of school-aged children in Ireland have low intakes of vitamin C and Zn (important for normal immune function); however, these DRV have been set based on evidence in adults and extrapolated to children and so should be interpreted with caution as there is currently no evidence of clinical manifestations for low vitamin C and Zn intakes in EU populations^(75)^. Additionally, EFSA have stated that further research is required to investigate vitamin C and Zn homoeostasis in children to further strengthen the evidence base for these recommendations and so while there is currently no evidence of deficiency, it is important to continue to monitor intakes among this population group.

This study has highlighted poor compliance with nutrient goals among school-aged children in Ireland which can be largely attributed to low compliance with the FBDG, including low consumption of wholemeal/high-fibre breads and cereals, fruit and vegetables and dairy foods and widespread consumption of SSB, confectionery and processed meats, which are not necessary for good health and are recommended to be consumed less frequently and in small amounts. As the dietary patterns developed at this young age have been shown to track into later life, it is important to address the challenges identified in this population group to reduce the prevalence of chronic diseases throughout the life cycle. Examination of other health determinant parameters among this population group highlights other challenges which need to be addressed for optimal lifestyle patterns for good health in children in Ireland. While the prevalence of overweight and obesity has plateaued since the previous NCFS rates remain high (16 %)^(82)^, and while a high proportion of this population group is meeting physical activity recommendations (69 %) there is still a need to reduce time spent in sedentary behaviours^(83)^. Furthermore, while over 78 % of parents felt their child’s diet could be healthier, they highlighted a number of barriers to implementing this including convenience and food advertising^(83)^. These findings suggest that there is a need for intersectoral policies and cross-collaboration between public health agencies for improving the dietary intakes of children in Ireland. Examples of these may include further investments in education around ‘healthy’ lifestyle patterns for all ages, food marketing restrictions (particularly related to advertisements targeted for young children) and policies for improvement of the food supply, for example, reformulation and fortification.

### Strengths and limitations

This is the first study to investigate changes in food and nutrient intakes in nationally representative samples of school-aged children in Ireland over a 15-year period. The main strengths of this study are the detailed dietary intake data (including brand-level detail on fortified foods and nutritional supplements and customised recipes) and the use of nationally representative data for estimating food and nutrient intakes and adequacy. The use of statistical modelling to estimate usual intakes accounts for day-to-day inter- and intra-person variation and results in a better estimate of the true distribution of nutrient intakes. It also improves the estimates of the proportions of the population with intakes above or below particular reference values (e.g. UL and EAR) which otherwise would be overestimated. While the food record completion time (7 *v*. 4 d) differed between the two surveys, this is unlikely to impact on mean intakes of foods or nutrients; however, any changes in the proportion of consumers should be interpreted with caution (particularly for foods consumed less frequently). As with all self-reported data, under- or over-reporting of food intake data may be sources of bias. This issue was minimised by a high level of researcher–participant interaction (3–4 visits over the recording period). Additionally, nutrient intakes were expressed as a percentage of energy intake (where appropriate) which partially corrects this bias and the removal of URs from estimates of the prevalence of inadequacy provides a better representation of the scale of nutrient inadequacy.

### Conclusion

In summary, while there have been some changes in the dietary intakes of school-aged children in Ireland over a 15-year period, including decreased intakes of SSB, fruit juice, milk and potatoes, and increased intakes of wholemeal/brown bread, high-fibre RTEBC, porridge, pasta and whole fruit, this population group still have higher intakes of saturated fat, free sugar and salt, and lower intakes of dietary fibre, vitamin D, Ca, Fe and folate compared with recommendations. Future strategies to address the nutrient gaps identified among this population group could include the continued promotion of healthy food choices (including education around ‘healthy’ lifestyles and food marketing restrictions), improvements of the food supply through reformulation (fat, sugar, salt, dietary fibre), food fortification for micronutrients of concern (voluntary or mandatory) and/or nutritional supplement recommendations (for nutrients unlikely to be sufficient from food intake alone). These strategies will require careful monitoring to ensure effectiveness, for example, reformulation and/or fortification of the food supply should be monitored to ensure sufficient bioavailability of nutrients and that these practices do not encourage consumption of ‘less healthy’ foods. With a global emphasis to update FBDG to incorporate environmental sustainability and sociocultural factors, the current study provides valuable information on the baseline/current dietary patterns of school-aged children in Ireland.

## Supplementary material

For supplementary material accompanying this paper visit https://doi.org/10.1017/S0007114522002781. click here to view supplementary material
