# Supplementary material for: Food and nutrient intakes and compliance with recommendations in school-aged children in Ireland: findings from the National Children’s Food Survey II (2017–2018) and changes since 2003–2004
Source: Br J Nutr. 2022 Sep 1;129(11):2011–24. doi: 10.1017/S0007114522002781 (PMC10167663; doi:10.1017/S0007114522002781)
Supplement: Supplementary file 1 [file S0007114522002781sup001.docx]

| **Supplementary Table 1.** Contribution (%) of food groups to mean daily intakes of energy, macronutrients and dietary fibre in children aged 5-12 years in the NCFS II (2017-18) | | | | | | | | | | | | | | | | | | | |
| --- | --- | --- | --- | --- | --- | --- | --- | --- | --- | --- | --- | --- | --- | --- | --- | --- | --- | --- | --- |
|  | Energy |  | Protein |  | Total Fat |  | Saturated Fat |  | MUFA |  | PUFA |  | Carbohydrate |  | Total Sugar |  | Free Sugar |  | Dietary Fibre |
|  | % contribution | | | | | | | | | | | | | | | | | | |
| Bread & rolls | 14.3 |  | 13.2 |  | 5.2 |  | 3.7 |  | 4.7 |  | 10.9 |  | 21.1 |  | 4.6 |  | 1.1 |  | 22.5 |
| Meat & meat products | 14.3 |  | 32.7 |  | 21.4 |  | 18.1 |  | 25.0 |  | 20.7 |  | 4.2 |  | 2.6 |  | 2.1 |  | 7.3 |
| Milk & yogurt | 10.1 |  | 13.8 |  | 12.5 |  | 18.5 |  | 10.4 |  | 2.7 |  | 7.6 |  | 19.3 |  | 10.2 |  | 0.9 |
| Breakfast cereals | 8.9 |  | 6.3 |  | 3.4 |  | 3.1 |  | 2.7 |  | 6.3 |  | 12.4 |  | 7.0 |  | 11.0 |  | 14.3 |
| Grains, rice, pasta & savouries | 8.7 |  | 8.2 |  | 5.7 |  | 5.3 |  | 5.4 |  | 7.8 |  | 11.0 |  | 1.6 |  | 0.7 |  | 9.5 |
| Sugars, confectionery, preserves & savoury snacks | 8.7 |  | 3.2 |  | 9.9 |  | 9.5 |  | 11.4 |  | 7.3 |  | 9.6 |  | 15.5 |  | 26.3 |  | 6.2 |
| Biscuits, cakes & pastries | 8.5 |  | 3.4 |  | 10.4 |  | 11.8 |  | 9.8 |  | 9.0 |  | 9.0 |  | 10.1 |  | 18.0 |  | 5.4 |
| Potatoes & potato products | 5.5 |  | 2.6 |  | 4.9 |  | 2.9 |  | 5.3 |  | 8.1 |  | 7.0 |  | 0.8 |  | 0.0 |  | 9.4 |
| Fruit & fruit juices | 5.0 |  | 1.8 |  | 0.7 |  | 0.5 |  | 0.4 |  | 2.0 |  | 8.8 |  | 22.0 |  | 10.0 |  | 11.1 |
| Creams, ice-creams & chilled desserts | 3.3 |  | 1.8 |  | 3.9 |  | 4.7 |  | 3.1 |  | 3.7 |  | 3.3 |  | 5.6 |  | 7.7 |  | 1.3 |
| Butter & spreading fats | 2.8 |  | 0.1 |  | 8.0 |  | 8.7 |  | 7.9 |  | 6.8 |  | 0.0 |  | 0.1 |  | 0.0 |  | 0.0 |
| Cheeses | 2.5 |  | 4.0 |  | 5.3 |  | 7.7 |  | 4.4 |  | 1.4 |  | 0.1 |  | 0.2 |  | 0.0 |  | 0.1 |
| Vegetables & vegetable dishes | 1.7 |  | 2.2 |  | 1.2 |  | 0.7 |  | 1.1 |  | 2.4 |  | 2.0 |  | 2.8 |  | 0.7 |  | 9.5 |
| Fish & fish dishes | 1.6 |  | 3.5 |  | 2.1 |  | 1.0 |  | 2.5 |  | 3.7 |  | 0.6 |  | 0.2 |  | 0.2 |  | 0.7 |
| Soups & sauces | 1.4 |  | 0.7 |  | 2.3 |  | 1.6 |  | 2.6 |  | 3.2 |  | 1.0 |  | 1.7 |  | 2.3 |  | 1.4 |
| Beverages | 1.3 |  | 0.2 |  | 0.1 |  | 0.1 |  | 0.0 |  | 0.0 |  | 2.3 |  | 5.8 |  | 9.6 |  | 0.2 |
| Eggs & egg dishes | 1.2 |  | 2.1 |  | 2.3 |  | 1.9 |  | 2.6 |  | 2.6 |  | 0.0 |  | 0.0 |  | 0.0 |  | 0.0 |
| Nuts, seeds, herbs & spices | 0.2 |  | 0.2 |  | 0.5 |  | 0.2 |  | 0.5 |  | 1.0 |  | 0.0 |  | 0.0 |  | 0.0 |  | 0.4 |
| Nutritional supplements | 0.0 |  | 0.0 |  | 0.1 |  | 0.0 |  | 0.1 |  | 0.3 |  | 0.0 |  | 0.0 |  | 0.1 |  | 0.0 |
| MUFA, monounsaturated fat; PUFA, polyunsaturated fat  **Note:** Food groups are listed in order of contribution to energy intakes (decreasing order) | | | | | | | | | | | | | | | | | | | |

| **Supplementary Table 2.** Contribution (%) of food groups to mean daily vitamin intakes in children aged 5-12 years in the NCFS II (2017-18) | | | | | | | | | | | | | | | | | | | | | | | | | |  |
| --- | --- | --- | --- | --- | --- | --- | --- | --- | --- | --- | --- | --- | --- | --- | --- | --- | --- | --- | --- | --- | --- | --- | --- | --- | --- | --- |
|  | Vit A |  | Vit D |  | Vit E |  | Thiamin |  | Riboflavin |  | Niacin |  | Vit B6 |  | Vit B12 |  | Folate |  | DFE |  | Biotin |  | Pantothenate |  | Vit C | |
|  | % contribution | | | | | | | | | | | | | | | | | | | | | | | | | |
| Bread & rolls | 1.1 |  | 1.0 |  | 3.1 |  | 14.7 |  | 4.9 |  | 12.2 |  | 5.9 |  | 0.6 |  | 13.7 |  | 12.4 |  | 6.6 |  | 7.3 |  | 0.2 | |
| Meat & meat products | 11.1 |  | 19.6 |  | 13.2 |  | 17.1 |  | 11.4 |  | 33.5 |  | 26.8 |  | 21.1 |  | 8.5 |  | 7.8 |  | 12.1 |  | 18.8 |  | 8.9 | |
| Milk & yogurt | 15.1 |  | 17.2 |  | 6.9 |  | 6.6 |  | 31.0 |  | 6.3 |  | 9.1 |  | 34.7 |  | 12.3 |  | 11.4 |  | 23.2 |  | 23.0 |  | 9.1 | |
| Breakfast cereals | 1.6 |  | 23.4 |  | 5.6 |  | 18.2 |  | 22.0 |  | 14.5 |  | 17.8 |  | 11.7 |  | 24.0 |  | 30.4 |  | 9.7 |  | 13.1 |  | 3.3 | |
| Grains, rice, pasta & savouries | 3.6 |  | 2.3 |  | 6.7 |  | 5.6 |  | 2.9 |  | 6.6 |  | 2.5 |  | 3.6 |  | 3.6 |  | 3.1 |  | 5.9 |  | 7.0 |  | 1.1 | |
| Sugars, confectionery, preserves & savoury snacks | 1.5 |  | 1.2 |  | 8.5 |  | 2.0 |  | 3.9 |  | 2.3 |  | 2.6 |  | 2.9 |  | 2.5 |  | 2.6 |  | 3.0 |  | 2.1 |  | 1.4 | |
| Biscuits, cakes & pastries | 3.8 |  | 1.9 |  | 10.1 |  | 3.1 |  | 2.1 |  | 3.3 |  | 1.7 |  | 1.3 |  | 2.9 |  | 2.5 |  | 6.8 |  | 3.4 |  | 0.2 | |
| Potatoes & potato products | 1.1 |  | 0.2 |  | 6.0 |  | 7.3 |  | 1.5 |  | 2.9 |  | 4.8 |  | 0.3 |  | 5.9 |  | 5.4 |  | 1.1 |  | 4.4 |  | 11.4 | |
| Fruit & fruit juices | 2.0 |  | 0.0 |  | 4.6 |  | 10.5 |  | 3.3 |  | 2.5 |  | 9.8 |  | 0.2 |  | 8.8 |  | 7.9 |  | 7.8 |  | 5.4 |  | 39.2 | |
| Creams, ice-creams & chilled desserts | 4.0 |  | 2.2 |  | 4.4 |  | 1.3 |  | 3.6 |  | 1.1 |  | 1.0 |  | 3.1 |  | 1.1 |  | 1.0 |  | 3.2 |  | 2.4 |  | 0.7 | |
| Butter & spreading fats | 7.4 |  | 3.1 |  | 5.0 |  | 0.0 |  | 0.3 |  | 0.0 |  | 0.5 |  | 0.6 |  | 0.4 |  | 0.6 |  | 0.1 |  | 0.0 |  | 0.0 | |
| Cheese | 7.0 |  | 1.8 |  | 1.0 |  | 0.3 |  | 2.7 |  | 2.4 |  | 1.0 |  | 5.0 |  | 1.6 |  | 1.4 |  | 2.2 |  | 1.1 |  | 0.0 | |
| Vegetables & vegetable dishes | 25.9 |  | 0.1 |  | 4.7 |  | 5.0 |  | 1.8 |  | 2.1 |  | 3.1 |  | 0.1 |  | 7.0 |  | 6.2 |  | 2.9 |  | 2.5 |  | 12.3 | |
| Fish & fish dishes | 0.5 |  | 7.0 |  | 4.5 |  | 1.4 |  | 1.0 |  | 3.4 |  | 1.7 |  | 5.2 |  | 0.8 |  | 0.7 |  | 1.4 |  | 1.5 |  | 0.4 | |
| Soups & sauces | 4.6 |  | 0.3 |  | 4.7 |  | 2.7 |  | 0.7 |  | 0.9 |  | 1.2 |  | 0.2 |  | 1.0 |  | 0.8 |  | 0.6 |  | 0.6 |  | 1.3 | |
| Beverages | 1.1 |  | 0.6 |  | 0.1 |  | 0.3 |  | 0.5 |  | 1.7 |  | 3.1 |  | 0.4 |  | 0.9 |  | 1.0 |  | 1.3 |  | 0.8 |  | 2.5 | |
| Eggs & egg dishes | 2.9 |  | 8.5 |  | 2.7 |  | 0.6 |  | 2.9 |  | 1.2 |  | 0.8 |  | 5.0 |  | 1.8 |  | 1.6 |  | 6.4 |  | 2.3 |  | 0.0 | |
| Nuts, seeds, herbs & spices | 0.1 |  | 0.0 |  | 0.4 |  | 0.2 |  | 0.1 |  | 0.3 |  | 0.2 |  | 0.0 |  | 0.2 |  | 0.1 |  | 1.0 |  | 0.1 |  | 0.0 | |
| Nutritional supplements | 5.7 |  | 9.8 |  | 8.1 |  | 3.0 |  | 3.5 |  | 2.9 |  | 6.1 |  | 4.0 |  | 2.8 |  | 3.2 |  | 4.5 |  | 4.1 |  | 8.0 | |
| Vit, Vitamin; DFE, Dietary Folate Equivalents | | | | | | | | | | | | | | | | | | | | | | | | | |  |
| **Note:** Food groups are listed in order of contribution to energy intakes (decreasing order) | | | | | | | | | | | | | | | | | | | | | | | | | |  |

| **Supplementary Table 3.** Contribution (%) of food groups to mean daily mineral intakes in children aged 5-12 years NCFS II (2017-18) | | | | | | | | | | | | | | | |
| --- | --- | --- | --- | --- | --- | --- | --- | --- | --- | --- | --- | --- | --- | --- | --- |
|  | Sodium |  | Potassium |  | Calcium |  | Iron |  | Magnesium |  | Zinc |  | Copper |  | Phosphorus |
|  | % contribution | | | | | | | | | | | | | | |
| Bread & rolls | 22.0 |  | 7.7 |  | 17.7 |  | 17.6 |  | 13.8 |  | 11.8 |  | 17.6 |  | 11.9 |
| Meat & meat products | 23.8 |  | 18.1 |  | 5.0 |  | 16.0 |  | 14.5 |  | 25.8 |  | 13.1 |  | 21.2 |
| Milk & yogurt | 6.7 |  | 17.7 |  | 34.9 |  | 1.5 |  | 13.9 |  | 15.4 |  | 4.1 |  | 21.2 |
| Breakfast cereals | 5.3 |  | 5.3 |  | 7.5 |  | 28.0 |  | 10.5 |  | 9.8 |  | 9.9 |  | 7.7 |
| Grains, rice, pasta & savouries | 7.1 |  | 4.0 |  | 6.5 |  | 6.6 |  | 7.6 |  | 8.1 |  | 10.2 |  | 7.3 |
| Sugars, confectionery, preserves & savoury snacks | 5.5 |  | 4.8 |  | 3.6 |  | 4.5 |  | 5.3 |  | 3.5 |  | 6.9 |  | 3.5 |
| Biscuits, cakes & pastries | 5.8 |  | 3.3 |  | 3.8 |  | 6.3 |  | 5.5 |  | 3.5 |  | 7.5 |  | 4.7 |
| Potatoes & potato products | 2.0 |  | 13.3 |  | 1.2 |  | 3.8 |  | 7.3 |  | 2.6 |  | 7.9 |  | 3.6 |
| Fruit & fruit juices | 0.4 |  | 11.6 |  | 2.2 |  | 3.2 |  | 7.5 |  | 1.3 |  | 9.3 |  | 2.3 |
| Creams, ice-creams & chilled desserts | 1.5 |  | 2.2 |  | 3.1 |  | 1.8 |  | 2.1 |  | 1.6 |  | 2.1 |  | 2.4 |
| Butter & spreading fats | 2.7 |  | 0.1 |  | 0.1 |  | 0.0 |  | 0.1 |  | 0.1 |  | 0.1 |  | 0.1 |
| Cheeses | 4.5 |  | 0.6 |  | 8.6 |  | 0.4 |  | 1.6 |  | 4.8 |  | 0.5 |  | 5.6 |
| Vegetables & vegetable dishes | 2.1 |  | 5.4 |  | 2.3 |  | 4.2 |  | 4.4 |  | 2.7 |  | 5.4 |  | 2.6 |
| Fish & fish dishes | 2.0 |  | 1.9 |  | 0.6 |  | 1.0 |  | 1.8 |  | 1.1 |  | 1.3 |  | 2.3 |
| Soups & sauces | 5.4 |  | 1.9 |  | 0.7 |  | 1.1 |  | 1.2 |  | 0.7 |  | 1.5 |  | 0.7 |
| Beverages | 1.5 |  | 1.3 |  | 0.8 |  | 0.2 |  | 1.4 |  | 3.7 |  | 0.5 |  | 0.8 |
| Eggs & egg dishes | 1.8 |  | 0.7 |  | 0.8 |  | 1.9 |  | 0.7 |  | 1.6 |  | 0.8 |  | 1.9 |
| Nuts, seeds, herbs & spices | 0.1 |  | 0.2 |  | 0.1 |  | 0.2 |  | 0.5 |  | 0.3 |  | 0.6 |  | 0.2 |
| Nutritional supplements | 0.0 |  | 0.0 |  | 0.6 |  | 1.7 |  | 0.5 |  | 1.7 |  | 0.8 |  | 0.0 |
| **Note:** Food groups are listed in order of contribution to energy intakes (decreasing order) | | | | | | | | | | | | | | | |
